# Assessing Treatment Efficacy for Dermatologic Immune-Related Adverse Events: A Systematic Review

**DOI:** 10.1016/j.xjidi.2025.100410

**Published:** 2025-09-02

**Authors:** Christian L. Bailey-Burke, Kristin A. Tissera, Lauren Baughman, Samantha A. Polly, Meenal Kheterpal

**Affiliations:** 1 Duke University School of Medicine, Durham, North Carolina, USA; 2 Department of Dermatology, University of Rochester Medical Center, Rochester, New York, USA; 3 Department of Dermatology, Duke University School of Medicine, Durham, North Carolina, USA

**Keywords:** Cancer treatment, Dermatologic immune-related adverse event (D-irAE), Immune checkpoint inhibitor (ICI), Immunotherapy, Level of evidence

## Abstract

The management of dermatologic immune–related adverse events (D-irAEs) remains inconsistent owing to variability in treatment responses and a lack of standardized guidelines. Establishing evidence-based recommendations is critical to improving patient outcomes and minimizing interruptions in immune checkpoint inhibitor therapy. This review aims to systematically evaluate the level of evidence for reported D-irAE treatments and provide insights to guide clinical decision making. Of the D-irAEs identified, those that were commonly reported include maculopapular, lichenoid, psoriasiform, immunobullous (including bullous pemphigoid), granulomatous, vitiligo, and potentially life-threatening erosive mucocutaneous conditions (such as erythema multiforme, Steven–Johnson syndrome, and toxic epidermal necrosis). Treatments ranged from corticosteroids (topical, oral, intravenous) to biologics (eg, omalizumab, rituximab) and oral immunomodulators (eg, methotrexate, apremilast), with topical and oral corticosteroids, along with apremilast, receiving the strongest evidence rating (3B). Although the strongest evidence supports corticosteroids, most treatments for D-irAEs lack robust validation owing to limitations in study type (ie, randomized control trials and prospective cohort studies). This underscores the importance of multidisciplinary approaches to optimize D-irAE management and support continuity in cancer care. Future research should prioritize randomized control trials and prospective cohort studies to aid in standardizing D-irAE treatment protocols to solidify treatment consensus.

## Introduction

Dermatologic immune-related adverse events (D-irAEs) are a well-described sequelae of immune checkpoint inhibitors (ICIs), therapies that have improved cancer outcomes (Chen et al, 2024). These D-irAEs, which may manifest as maculopapular, psoriasiform, immunobullous, or granulomatous eruptions, can impact QOL and lead to treatment interruptions. More recently, an increased number of patients have become eligible for ICI therapy, which has resulted in a rise in the incidence of D-irAEs (Chen et al, 2024). Such an impact led to an international effort among oncologists, dermatologists, and other subspecialists to provide guidance for an approach to evaluating D-irAEs, including disease definitions and severity grading through a Delphia consensus process (Chen et al, 2024).

Despite these advances, consensus has yet to be reached on the optimal treatment for each D-irAE owing to response variability and the absence of robust, standardized guidelines. Ultimately, this often results in clinician reliance on anecdotal evidence in case reports to guide management. A previous systematic review by Nadelmann et al (2022) further categorized each D-irAE and outlined treatments ranging from systemic corticosteroids to immunomodulators (eg, methotrexate). However, inconsistent treatment responses further support the need for improved strategies to address these D-irAEs. This systematic review will build on these studies by providing a comprehensive evaluation of the evidence strength that supports the use of reported treatments in managing D-irAEs.

## Results

**Figure 1:**
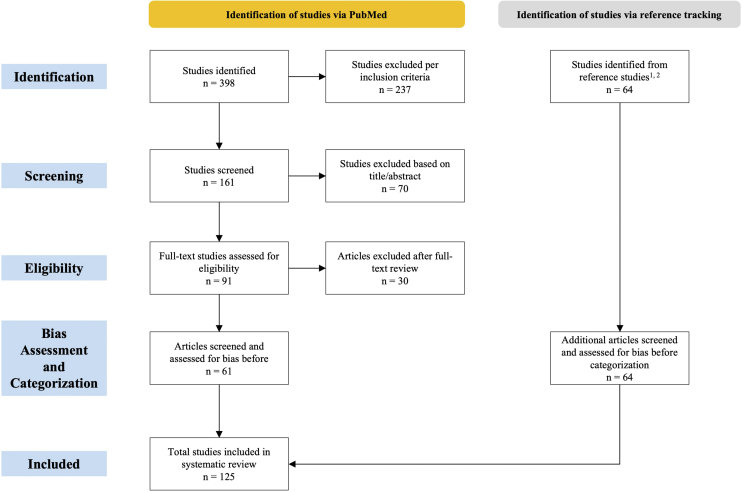
**Systematic review process overview.** Systematic review was conducted in alignment with PRISMA standards, including the number of studies identified, screened, and determined eligible. Study bias was assessed as part of study screening prior to study categorization. PRISMA, Preferred Reporting Items for Systematic reviews and Meta-Analyses.

The initial search in PubMed yielded a total of 398 articles. A total of 237 studies were excluded because they did not meet the inclusion criteria. Of the 161 studies screened, 70 were further excluded per the title and abstract context not meeting the scope of this review. Full-text review was performed for 91 studies to assess further eligibility, with 30 excluded at the full-text review stage. Sixty-one articles were assessed for bias and were categorized. Sixty-four additional studies were identified through reference tracking and further screened and assessed for bias prior to categorization. A total of 125 studies were included (Figure 1).

### Study data

Of the study design types reviewed, 6 were prospective, and 119 were retrospective, with 1015 D-irAE patient cases reported in total. The most commonly reported ICIs occurred with anti–PD-1 or PD-1 inhibitor (ie, nivolumab, pembrolizumab, cemiplimab), anti–PD-L1 or PD-L1 inhibitor (ie, atezolizumab, durvalumab, avelumab), and CTLA-4 or CTLA-4 inhibitor (ie, ipilimumab) therapies, with the majority of cases involving the use of anti–PD-1 therapy. D-irAEs reported fell into the following diagnosis categories per previously reported guidelines: maculopapular, psoriasiform, lichenoid, immunobullous, granulomatous, and vitiligo, along with rarer and more severe D-irAEs, including erythema multiforme, Steven–Johnson syndrome (SJS), and toxic epidermal necrosis (TEN), which were grouped under erosive mucocutaneous conditions (Chen et al, 2024).

In analyzing the type of therapies that triggered severe D-irAEs, all cases of SJS and TEN (17 patient cases in total) were due to PD-1 inhibitors (ie, nivolumab, pembrolizumab), which were most commonly used in patients with lung cancer and melanoma. Five drug reaction with eosinophilia and systemic symptoms cases were reported; 4 patients received PD-1 inhibitors (nivolumab, pembrolizumab) for melanoma, and 1 patient received a CTLA-4 inhibitor (ipilimumab) for melanoma. Four erythema multiforme cases were reported; 2 patients were on a PD-1 inhibitor for unspecified cancer, 1 patient was on a PD-L1 inhibitor for esophageal cancer, and 1 patient was on a CTLA-4 inhibitor for melanoma.

Time to D-irAE onset ranged significantly from 1 week to 18 months. Treatments initiated in the management of these D-irAEs ranged from topical corticosteroids to biologics (eg, rituximab), with a majority of cases reporting D-irAE improvement. Reported time to follow-up after treatment varied but ranged between 4 weeks and 2 years.

### Common D-irAEs: treatments and level of evidence

Of the 125 articles reviewed, 43 (34.4%) were identified as providing the highest level of evidence (LOE) supporting treatments for the most commonly reported D-irAEs (Table 1, Table 2, Table 3, Table 4, Table 5). It is important to note that these 43 studies were not necessarily limited to a single D-irAE or treatment (eg, multiple types of D-irAEs and their corresponding treatments were discussed in the case series; hence, the same article may be reflected across the 5 common D-irAEs reported below). Moreover, the Oxford ratings that were assigned to treatments were study-type dependent, with treatments mentioned in case-control studies receiving the highest LOE (3B), followed by case series (4), and then case reports (5). As a result, corticosteroids typically received the highest rating owing to the strongest evidence that supports their use compared with other treatments. Moreover, most D-irAEs improved with corticosteroid treatment.

**Table 1:** Maculopapular D-irAE Treatment and Level of Evidence

Treatment Category	Level of Evidence
Corticosteroid	
Topical	3B Min Lee et al, 2018
Oral	
Intravenous	4 Coleman et al, 2019
Phototherapy	
nb-UVA/B	4 Coleman et al, 2019; Shi et al, 2016; Shen et al, 2018
Immunomodulator	
Cyclosporine	5 Cai et al, 2020
Apremilast	4 L’Orphelin et al, 2023
Methotrexate	5 Ványai et al, 2022
Retinoid^1^	5 Mullangi et al, 2021
Antibiotic	
Oral (eg, tetracycline)	4 L’Orphelin et al, 2023
Biologic	
Omalizumab	4 Barrios et al, 2021
Other (eg, rituximab, dupilumab, ustekinumab)	4 Phillips et al, 2019
Topical antipruritic	
Camphor menthol	4 Coleman et al, 2019
Oral antipruritic	
Antihistamines H1/H2	4 L’Orphelin et al, 2023
Gabapentin	4 Coleman et al, 2019
Pregabalin	4 Lomax et al, 2018

Abbreviations: D-irAE, dermatologic immune–related adverse event; nb, narrow-band.

This table presents a summary for maculopapular D-irAEs, including treatments used across studies and assigned Oxford level of evidence for affiliated studies.

1 Retinoids were mentioned, but specific retinoid names not provided.

**Table 2:** Psoriasiform D-irAE Treatment and Level of Evidence

Treatment Category	Level of Evidence
Corticosteroid	
Topical	4 L’Orphelin et al, 2023
Oral	
Other (eg, intralesional Kenalog)	4 Coleman et al, 2019
Phototherapy	
nb-UVB	4 Coleman et al, 2019; Shen et al, 2018; Voudouri et al, 2017
Immunomodulator	
Apremilast	4 L’Orphelin et al, 2023
Methotrexate	4 Voudouri et al, 2017; Coleman et al, 2019
Other (eg, mycophenolate)	4 Phillips et al, 2019
Retinoid^1^	4 Coleman et al, 2019; Mayor Ibarguren et al, 2021
Antibiotic	
Topical (eg, mupirocin)	4 Totonchy et al, 2016
Other (eg, unspecified)	4 Rovers et al, 2020
Biologic	
Secukinumab	5 Monsour et al, 2019
Vitamin	
Topical calcipotriene	4 Hashimoto et al, 2021; Voudouri et al, 2017
Topical antipruritic	
Camphor menthol	4 Coleman et al, 2019
Oral antipruritic	
Antihistamines H1/H2	4 L’Orphelin et al, 2023
Gabapentin	4 Coleman et al, 2019
Pregabalin	4 Phillips et al, 2019
Other (eg, aprepitant)	

Abbreviations: D-irAE, dermatologic immune–related adverse event; nb, narrow-band.

This table presents a summary for psoriasiform D-irAEs, including treatments used across studies and assigned Oxford level of evidence for affiliated studies.

1 Retinoids were mentioned, but specific retinoid names not provided.

**Table 3:** Lichenoid D-irAE Treatment and Level of Evidence

Treatment Category	Level of Evidence
Corticosteroid	
Topical	3B Min Lee et al 2018
Oral	
Intravenous	4 Chou et al, 2017; Coleman et al, 2019; Goldinger et al, 2016; Hashimoto et al, 2021
Other (eg, intralesional Kenalog)	4 Coleman et al, 2019
Phototherapy	
nb-UVB	4 Coleman et al, 2019
Immunomodulator	
Cyclosporine	5 Fixsen et al, 2019
Apremilast	4 L’Orphelin et al, 2023
Hydroxychloroquine	5 Coscarart et al, 2020; Uthayakumar et al, 2021
Intravenous Ig	4 Hashimoto et al, 2021
Other (eg, Mycophenolate)	4 Phillips et al, 2019
Retinoid^1^	4 Coleman et al, 2019; Uthayakumar et al, 2021
Antibiotic	
Oral (eg, tetracycline)	4 Uthayakumar et al, 2021
Biologic	
Omalizumab	5 Marques-Piubelli et al, 2020
Other (eg, rituximab)	4 Phillips et al, 2019
Vitamin	
Oral nicotinamide	4 Star et al, 2022
Oral vitamin D_3_	4 Hashimoto et al, 2021
Chemotherapy	
5-Fluorouracil	5 Ameri et al, 2020,
Topical antipruritic	
Camphor menthol	4 Coleman et al, 2019
Oral antipruritic	
Antihistamines H1/H2	4 L’Orphelin et al, 2023
Gabapentin	4 Coleman et al, 2019; Phillips et al, 2019,
Pregabalin	4 Phillips et al, 2019

Abbreviations: D-irAE, dermatologic immune–related adverse event; nb, narrow-band.

This table provides a summary for lichenoid D-irAEs, including treatments used across studies and assigned Oxford level of evidence for affiliated studies.

1 Retinoids were mentioned, but specific retinoid names not provided.

**Table 4:** Other Autoimmune D-irAEs Treatment and Level of Evidence

Treatment Category	Level of Evidence		
	Immunobullous	Vitiligo	Granulomatous
Corticosteroid			
Topical	3B Min Lee et al, 2018		4 Coleman et al, 2019; Dimitriou et al, 2018; Kaunitz et al, 2017
Oral	3B Min Lee et al, 2018		4 Dimitriou et al, 2018; Kaunitz et al, 2017
IV	4 Hashimoto et al, 2021		4 Reddy et al, 2017
Other (eg, intralesional Kenalog)	5 Ameri et al, 2020		
Immunomodulator			
Cyclosporine	5 Cai et al, 2020		
Apremilast	3B Min Lee et al, 2018		
Methotrexate	4 Kaunitz et al, 2017; Lopez et al, 2018		
IV Ig	5 Wu et al, 2022; Yang et al, 2021	4 Hashimoto et al, 2021,	
Dapsone	4 Coleman et al, 2019; Kaunitz et al, 2017		
TNF-α inhibitor	5 Wu et al, 2022,		
Retinoid	5 Lindner et al, 2018		
Antibiotic			
Oral (eg, tetracyclines)	4 Coleman et al, 2019; Hwang et al, 2016; Lopez et al, 2018		
Biologic			
Omalizumab	4 Barrios et al, 2021; Coleman et al, 2019		
Rituximab	5 Sharma et al, 2020; Sowerby et al, 2017; Virgen et al, 2020		
Vitamin			
Oral nicotinamide	4 Coleman et al, 2019,		
Oral vitamin D_3_		4 Hashimoto et al, 2021,	
Oral antipruritic			
Antihistamines H1/H2	4 Hwang et al, 2016; Lomax et al, 2018; Lopez et al, 2018; Phillips et al, 2019; Rovers and Bovenschen, 2020	4 Bonigen et al, 2017; Hashimoto et al, 2021; Hofmann et al, 2016; Lomax et al, 2018; Phillips et al, 2019; Rovers and Bovenschen, 2020; Voskens et al, 2013	
Pregabalin	4 Lomax et al, 2018		

Abbreviations: D-irAE, dermatologic immune–related adverse event; IV, intravenous.

This table provides a summary for other autoimmune D-irAEs, including immunobullous, vitiligo, and granulomatous, along with treatments used across studies and assigned Oxford level of evidence for affiliated studies.

**Table 5:** Erosive Mucocutaneous D-irAE Treatment and Level of Evidence

Treatment Category	Level of Evidence
Corticosteroid	
Topical	4 Coleman et al, 2019; Dika et al, 2017; Hashimoto et al, 2021
Oral	4 Coleman et al, 2019; Dika et al, 2017; Hashimoto et al, 2021; Naqash et al, 2019; Saw et al, 2017
IV	4 Hashimoto et al, 2021; Naqash et al, 2019
Immunomodulator	
Cyclosporine	4 Saw et al, 2017
IV Ig	4 Hashimoto et al, 2021
Biologic	
Infliximab	4 Coleman et al, 2019
Vitamin	
Oral vitamin D_3_	4 Hashimoto et al, 2021
Oral antipruritic	
Antihistamines H1/H2	4 Dika et al, 2017; Hashimoto et al, 2021

Abbreviations: D-irAE, dermatologic immune–related adverse event; IV, intravenous.

Table presents a summary for erosive mucocutaneous D-irAEs, including treatments used across studies and assigned Oxford level of evidence for affiliated studies.

#### Maculopapular

Of the 25 studies that reported cases of maculopapular D-irAEs, 11 (44.0%) were arranged by treatment and assigned a LOE (Table 1). Treatments for maculopapular D-irAEs included corticosteroids (ie, intravenous [IV], oral, topical), UVA/narrow-band UVB, methotrexate, cyclosporine, apremilast, omalizumab, ustekinumab, camphor menthol, and gabapentin. The strongest evidence supports the usage of corticosteroids, specifically oral and topical regimens (3B), followed by IV steroids for maculopapular D-irAE (4) (Coleman et a., 2019; MinLee et al, 2018). Among the immunomodulators, apremilast had the strongest evidence supporting its usage (4) than both cyclosporine and methotrexate (5) (Cai et al, 2020; L’Orphelin et al, 2023; Ványai et al, 2022). For biologics captured, omalizumab and others (eg, ustekinumab) had a rating of 4 (Barrios et al, 2021; Phillips et al, 2019). In reviewing the topical and oral antipruritic agents, including camphor menthol, antihistamines H1/H2, gabapentin, and pregabalin, the highest evidence that supports their use is a 4 (Coleman et al, 2019; Lomax et al, 2018; L'Orphelin et al, 2023). One treatment that was used in conjunction with others or separate depending on the patient was narrow-band UVA/B, which received a rating of 4 (Coleman et al, 2019; Shen et al, 2018; Shi et al, 2016). Finally, retinoids were a treatment utilized in the management of maculopapular D-irAE, but the evidence supporting their use was a 5 (Mullangi et al, 2021).

#### Psoriasiform

Twenty-four studies discussed psoriasiform D-irAEs, and 10 (41.7%) underwent additional analysis to assess the LOE with each reported treatment (Table 2). Treatment options were similar to those for maculopapular with the addition of secukinumab, aprepitant, and topical calcipotriene. The strongest evidence supporting the use of corticosteroids, specifically topical, oral, and intralesional regimens, in the management of psoriasiform D-irAEs is a 4 (Coleman et al, 2019; L'Orphelin et al, 2023). Within the immunomodulator class, apremilast, methotrexate, and other medications (eg, mycophenolate) received equivalent ratings (4) (Colemanet al, 2019; L'Orphelinet al, 2023; Mullangiet al, 2021; Voudouriet al, 2017). Retinoids had a rating of 4 (Colemanet al, 2019; Mayor Ibargurenet al, 2021). Of the vitamins used in the management of psoriasiform D-irAEs, topical calcipotriene received a rating of 4 (Hashimoto et al, 2021; Voudouri et al, 2017). Phototherapy was also reported, specifically narrow-band UVB, and was rated as a 4 (Coleman et al, 2019; Shen et al, 2018; Voudouri et al, 2017). Regarding topical and oral antipruritics, the evidence supporting their use, including aprepitant, was a 4 (Coleman et al, 2019; L'Orphelin et al, 2023; Phillips et al, 2019). Some less common medications reported from the studies included biologics such as secukinumab and topical antibiotics, both of which received LOE ratings of 5 (Monsour et al, 2019; Totonchy et al, 2016).

#### Lichenoid

A total of 36 studies involving patients who had experienced lichenoid D-irAEs (ie, lichen planus) were found, with 13 (39.4%) further categorized by treatments (Table 3). Treatments for this D-irAE type are inclusive of those used in the management of maculopapular and psoriasiform D-irAEs. However, other treatments reported effective include nicotinamide and hydroxychloroquine (Table 3).

Of the steroid treatments, oral and topical corticosteroids received the strongest rating (3B) for use in the management of lichenoid D-irAEs (MinLee et al, 2018). This was followed by intralesional Kenalog and IV corticosteroids, which were both rated at a 4 (Chou et al, 2017; Coleman et al, 2019; Goldinger et al, 2016; Hashimoto et al, 2021). Among the other therapy classes, most received a rating of a 4, including phototherapy (narrow-band UVB), immunomodulators (apremilast, IV Ig), retinoids, oral antibiotics, vitamins (nicotinamide and vitamin D_3_), and topical and oral antipruritics (ie, camphor menthol, gabapentin) (Coleman et al, 2019; Hashimoto et al, 2021; L'Orphelin et al, 2023; Phillips et al, 2019; Star et al, 2022). Therapies that were associated with a level 5 include hydroxychloroquine, 5-fluorouracil, and omalizumab (Ameri et al, 2020; Coscarart et al, 2020; Marques-Piubelli et al, 2020; Uthayakumar et al, 2021).

#### Other autoimmune

For immunobullous, of the 36 articles identified describing immunobullous D-irAEs (ie, bullous pemphigoid), 19 (52.8%) were further categorized by treatment and assigned a LOE (Table 4 [immunobullous]). The most common treatments include corticosteroids (eg, IV, oral, topical), immunomodulators (ie, IV Ig, methotrexate, dapsone), and biologics (ie, omalizumab, rituximab). Other treatments that were less commonly reported yet mentioned as effective were retinoids, oral antibiotics, oral nicotinamide, and topical and oral antipruritics.

Per the Oxford LOE rating, the strongest evidence supports the use of corticosteroids, specifically oral and topical regimens (3B), followed by intralesional and IV steroids for immunobullous conditions (4, 5) (Ameri et al, 2020; Hashimoto et al, 2021; MinLee et al, 2018). Of the immunomodulators used in the studies, apremilast also received the strongest rating (3B), whereas methotrexate and dapsone received a rating below this (4) (Coleman et al, 2019; Kaunitz et al, 2017; Lopez et al, 2018; Min Lee et al, 2018). Other therapies in this class, including cyclosporine and IV Ig, received a 5 (Cai et al, 2020; Wu et al, 2022; Yang et al, 2021). Retinoids received a 5 (Lindner et al, 2018). For oral antibiotics, the highest rating received was a 4 (Coleman et al, 2019; Hwang et al, 2016; Lopez et al, 2018). In the biologics class, omalizumab received the highest rating (4), with rituximab receiving the lowest (5) (Barrios et al, 2021; Coleman et al, 2019; Sharma et al, 2020; Sowerby et al, 2017; Virgen et al, 2020). Among the vitamins reported, oral nicotinamide, which was used in conjunction with oral antibiotics, received a rating of a 4 (Coleman et al, 2019). Finally, among oral antipruritics, including antihistamine H1/H2 receptor blockers and pregabalin, the highest evidence supporting their use is a 4 (Hwang et al, 2016; Lomax et al, 2018; Lopez et al, 2018; Phillips et al, 2019; Rovers and Bovenschen, 2020).

#### Vitiligo

A total of 16 studies mentioning vitiligo were identified, with 9 (56.3%) extracted and assigned a LOE per treatment (Table 4 [vitiligo]). The most common treatments included IV, oral, and topical corticosteroids, followed by oral antipruritics. Less common agents were antibiotics, antifungals, biologics, and immunomodulators. Of the steroids, oral and topical regimens received the highest rating (3B), followed by IV (4) (Hashimoto et al, 2021; Min Lee et al, 2018). Of the immunomodulators, apremilast received a 3B (Min Lee et al, 2018). Additional therapies in this class, including IV Ig, received a 4 (Hashimoto et al, 2021). Of the antipruritics, antihistamine H1/H2 receptor blockers and pregabalin received a 4 (Hashimoto et al, 2021; Hofmann et al, 2016; Lomax et al, 2018; Phillips et al, 2019; Rovers and Bovenschen, 2020; Voskens et al, 2013). Finally, oral vitamin D_3_ received a 4 (Kaunitz et al, 2017).

#### Granulomatous

Eight studies reported granulomatous D-irAE (eg, sarcoidosis), with 4 (50%) screened for treatments with the highest LOE (Table 4 [granulomatous]). Of the reported treatments, only IV, oral, and topical steroids were found effective. Moreover, the strength of evidence that supports their use in the management of granulomatous eruptions is a 4 (Coleman et al, 2019; Dimitriou et al, 2018; Kaunitz et al, 2017; Reddy et al, 2017).

#### Erosive mucocutaneous

In the final D-irAE category, 23 studies mentioned the more severe D-irAEs or erosive mucocutaneous D-irAEs, including erythema multiforme, SJS, TEN, and variations (eg, SJS like, TEN like), with 5 studies (21.7%) proceeding to the next phase of analysis (Table 5 [erosive mucocutaneous]). The most common treatment found for these D-irAEs included corticosteroids (ie, IV, oral, and topical). Other therapies used in erosive mucocutaneous D-irAE management were cyclosporine, IV Ig, infliximab, oral vitamin D_3_, and antipruritics of the antihistamine H1/H2 receptor blocker class. The highest evidence rating for all the treatments was a 4 (Coleman et al, 2019; Dika et al, 2017; Hashimoto et al, 2021; Naqash et al, 2019; Saw et al, 2017).

## Discussion

On the basis of the findings from our systematic review, oral and topical corticosteroids received the highest Oxford LOE rating (3B) for most of the D-irAEs, including lichenoid and maculopapular reactions. Their high utilization is consistent with prior studies that have reported significant symptom improvement with corticosteroid usage (Siegel et al, 2018). Of the other therapies, immunomodulators (eg, cyclosporine, apremilast) and biologics (eg, omalizumab) demonstrated potential efficacy in reported patient cases, yet the evidence to support their use is limited, which is consistent with a prior analysis by Quach et al (2021), among other studies, who highlighted the need for subsequent studies to validate other emerging therapies (Barrios et al, 2021). Thus, these results indicate that despite the existence of novel treatments, there is a continued reliance on corticosteroids as first-line therapy.

Our analysis also demonstrates that the treatment efficacy and the evidence that supports it vary across different D-irAEs. For instance, corticosteroids were the cornerstone of treatment for maculopapular, lichenoid, immunobullous, and vitiligo D-irAEs, supported by an LOE rating of 3B. For lichenoid D-irAEs, intralesional Kenalog and narrowband UVB phototherapy offer additional therapeutic options in more refractory cases (Shi et al, 2016). For immunobullous D-irAE, corticosteroids are effective, although other immunomodulators, such as IV Ig and rituximab, are often required for severe or steroid-refractory cases (Lopez et al, 2018). Severe D-irAEs, such as SJS and TEN, are rare, and hence, fewer studies have reported the treatments in steroid-refractory cases. Moreover, as mentioned previously, studies have shown that these severe D-irAEs tend to be associated more with PD-1 therapy than with PD-L1 and CTLA-4 therapies (Satoh et al, 2024). Smaller studies have indicated that immunomodulators, such as cyclosporine and infliximab, have potential therapeutic benefit, but larger multi-institutional studies focusing on severe D-irAEs are needed to further investigate this. Collectively, these findings further show corticosteroids’ role in D-irAE management but highlight the need for more tailored approaches for severe or refractory cases, which may require a multidisciplinary approach involving both dermatology and oncology teams (Jacoby et al, 2023).

However, there are a few limitations to our study. First, there is heterogeneity in study designs. The studies in this systematic review primarily comprised retrospective case series and case reports, with only 6 prospective studies of the 125 total studies included in the analysis. This potentially limits their generalizability and further complicates the synthesis of robust treatment guidelines (Lopez et al, 2018). In addition, the absence of randomized control trials (RCTs) makes it difficult to directly compare efficacy across treatments. Thus, despite the advances in D-irAE management, there are significant gaps regarding the optimal approach for treatment management owing to limited higher-order evidence (Oxford LOE ratings of 1 and 2). Moreover, with the emergence of therapies such as Jak inhibitors and dupilumab, it is important to determine how to address these gaps to better guide therapeutic choices and patient prognosis (Hirotsu et al, 2022).

Future research should focus on conducting RCTs and prospective cohort studies to further validate the efficacy of new therapies, especially those that can target multiple underlying morphologies such as eczematous, lichenoid, psoriasiform, etc. One such class is Jak inhibitors (abrocinitib, tofacitinib), perhaps even eliminating the need for differentiation between etiologies and accelerating treatment responses by the oncology teams. However, given the immunosuppressive qualities of Jak inhibitors, safety in populations living with cancer remains to be determined through prospective studies. Additional studies are needed to establish comparative benchmarks, which can help strengthen the LOE for D-irAE treatments mentioned earlier. Furthermore, Delphi-consensus studies expanding real-world data registries could be leveraged for dermatology and oncology experts to align on evidence-based standardized management protocols, with the goal of improving QOL for our patients (Deutsch et al, 2023).

The evidence supporting D-irAE treatments remains limited, with corticosteroids being the most reliable therapy. As a result of the LOE variability and lack of expert consensus around optimal D-irAE management, further research, including RCTs and prospective cohort studies, is needed to advance patient care.

## Materials and Methods

### Identification

Inclusion criteria for this review included original investigations, case-control studies, case reports, and case series that discussed cutaneous immune-related adverse events (D-irAEs) associated with ICIs and published in English. A PubMed search from January 1, 2010 to January 4, 2024 used the terms ‘checkpoint inhibitor immunotoxicity,’ ‘cutaneous drug reaction,’ and ‘cutaneous immune-related adverse events.’ Additional studies were manually identified through reference tracking (Chen et al, 2024; Nadelmann et al, 2022). Figure 1 provides a tailored PRISMA (Preferred Reporting Items for Systematic reviews and Meta-Analyses) diagram.

### Screening

Three authors (LB, MK, and SAP) independently screened the articles by title and abstract. Discordance resulted in a full-text review by both screeners. At the full-text review stage, articles were categorized into 4 groups: case series with and without discussion of treatment and case reports with and without discussion of treatment. Original investigations and case-control studies were not categorized into these groups (Figure 1). Two authors (CLB and KAT) independently assessed bias in original investigations, case-control studies, case series, and case reports using the Joanna Briggs Institute Critical Appraisal Checklists (Joanna Briggs Institute, 2020).

### Data extraction

Data from eligible studies were organized into an Excel spreadsheet with predetermined fields, including but not limited to the following: study type (ie, retrospective or prospective), number of D-irAE patient cases, ICI drug used, D-irAE type, time to onset of D-irAE, D-irAE treatment used, and D-irAE treatment outcome. Treatments discussed for the most common D-irAEs were further grouped and organized into 5 groups (Table 1, Table 2, Table 3, Table 4, Table 5) and assigned an LOE per the Oxford Centre for Evidence-Based Medicine (March 2009) guidelines (Phillips et al, 2009). In this way, studies with the highest quality of evidence (eg, individual RCTs) were assigned a level 1, whereas studies with the lowest quality of evidence (eg, case series or case-control studies, expert opinion) were assigned a level 4 and level 5, respectively. Generally, high-quality RCTs are prioritized, but if evidence is lacking, the system moves down the hierarchy to the best evidence available.

## Data Availability Statement

Data and materials that support the findings in this study are available within the Duke University Box folder and can be accessed through the following link: https://duke.app.box.com/s/zv3z6twfne6gx1xddkut5vw10zanxk9l.

## ORCIDs

Christian L. Bailey-Burke: http://orcid.org/0000-0002-3988-9519

Kristin A. Tissera: http://orcid.org/0000-0003-0317-4156

Lauren Baughman: http://orcid.org/0009-0004-6748-0229

Samantha A. Polly: http://orcid.org/0009-0009-3870-4226

Meenal Kheterpal: http://orcid.org/0000-0002-0460-6400

## Conflict of Interest

The authors state no conflict of interest.
